# Regulation of Milk Protein Synthesis by Free and Peptide-Bound Amino Acids in Dairy Cows

**DOI:** 10.3390/biology10101044

**Published:** 2021-10-14

**Authors:** Miaomiao Zhou, Lianbin Xu, Fengqi Zhao, Hongyun Liu

**Affiliations:** 1 Key Laboratory of Molecular Animal Nutrition Ministry of Education, College of Animal Science, Zhejiang University, Hangzhou 310029, China; zhoumiaomiao@lcu.edu.cn (M.Z.); lianbinxu@zju.edu.cn (L.X.); 2 College of Agriculture, Liaocheng University, Liaocheng 252000, China; 3 Department of Animal and Veterinary Sciences, University of Vermont, Burlington, VT 05405, USA

**Keywords:** amino acids, peptides, lactation, milk production, signaling pathway

## Abstract

**Simple Summary:**

Free and peptide-bound amino acids are the main substrates for milk protein synthesis in the mammary gland. The milk protein concentration and yield of dairy cows are regulated by free and peptide-bound amino acids. The present article reviews the effects of free and peptide-bound amino acid supply on milk protein synthesis and their underlying mechanisms.

**Abstract:**

Milk protein (MP) synthesis in the mammary gland of dairy cows is a complex biological process. As the substrates for protein synthesis, amino acids (AAs) are the most important nutrients for milk synthesis. Free AAs (FAAs) are the main precursors of MP synthesis, and their supplies are supplemented by peptide-bound AAs (PBAAs) in the blood. Utilization of AAs in the mammary gland of dairy cows has attracted the great interest of researchers because of the goal of increasing MP yield. Supplying sufficient and balanced AAs is critical to improve MP concentration and yield in dairy cows. Great progress has been made in understanding limiting AAs and their requirements for MP synthesis in dairy cows. This review focuses on the effects of FAA and PBAA supply on MP synthesis and their underlying mechanisms. Advances in our knowledge in the field can help us to develop more accurate models to predict dietary protein requirements for dairy cows MP synthesis, which will ultimately improve the nitrogen utilization efficiency and lactation performance of dairy cows.

## 1. Introduction

Milk has important nutritional properties that are beneficial to the health and growth of infants [1,2]. In addition, milk, especially bovine milk, is an important source of essential nutrients in human diet. Milk protein (MP) can provide essential amino acids (EAAs) and has high nutritional value. Amino acids (AAs) are the building blocks of protein synthesis; they also suppress protein catabolism and serve as substrates for gluconeogenesis [2,3]. Furthermore, milk also contains many bioactive proteins [4].

Most MPs are mammary-derived, synthesized within mammary epithelial cells (MECs). Mammary-derived MPs consist of casein and whey proteins [5,6]. Casein accounts for approximately 80% of the MPs in dairy cows [6,7]. Mammary-derived MPs are synthesized using substrates extracted from blood as free AAs (FAAs) and peptide-bound AAs (PBAAs). MP synthesis and secretion is a complex biological process, involving integrated steps such as FAA and PBAA uptake, transcription and translation of MP genes, proteins modification after translation, and finally, secretion of the proteins into the alveolar lumen [8,9,10,11].

In consideration of the increasing demand for milk quality by consumers and the economic benefits of producers, there is an urgent need for the development of the dairy industry to improve MP concentration and yield. The change of MP concentration may be related to hormones (particularly prolactin, hydrocortisone, insulin, and growth hormone), nutrient (AA and energy) availability, and environmental stresses [12,13,14,15,16]. Dietary manipulations are a method for rapidly improving MP concentration and yield [13,14,15]. Among dietary nutrients, the amount and balance of AAs are the most important factors for MP synthesis [14,15]. Thus, absorption and utilization of FAAs and PBAAs by the mammary gland (MG) can regulate MP synthesis. The present review will focus on the regulation of MP synthesis by FAA and PBAA in dairy cows.

## 2. Determination of Limiting AAs

The state of an animal’s nutrition depends on the AA and energy supply [15]. Knowledge about the AA supply and requirements is required to predict MP yields. In NRC (2001), according to the studies of Schwab (1996) and Rulquin et al. (1993), AA requirements for MP synthesis of dairy cows were recommended for methionine (Met) and lysine (Lys) [17,18,19]. Met and Lys are limiting AA for ruminants in certain diets. The maximum MP content and yield could be obtained by 7.2% of Lys and 2.4% Met in the diet. The study of Wang et al. (2010) also demonstrated that when the ratio of Lys to Met was 3:1, nitrogen (N) utilization efficiency and MP synthesis could be improved to the greatest extent [20].

In addition to Met and Lys, other EAAs may also be potentially limiting for MP synthesis. For example, the addition of rumen-protected histidine (His, 7 g/d of digestible His) in diets containing 5% hydrolyzed feather meal increased milk yield by 4.2% and tended to increase MP yield by 3.8% of dairy cows [21]. His may be limiting for diets containing hydrolyzed feather meal [21]. Recent studies have evaluated arginine (Arg), phenylalanine (Phe), isoleucine (Ile), leucine (Leu), valine (Val), and threonine (Thr), His as potentially limiting AA for MP synthesis [21,22,23,24]. These individual AAs affect the rates of MP synthesis in the MG of lactating cows. One study quantified the effect of Met, Leu, Ile, and Thr addition on the α_s1_-casein fractional synthesis rate (CFSR) to investigate the single limiting AA theory for milk production (a single EAA limits MP yield) [25]. The results showed that the responses of CFSRs to Met, Leu, Ile, and Thr were independent, which contradicts with the single limiting AA theory [25]. In the research of Yoder et al. (2020), the production responses of dairy cows to two groups, with AAs, Met, Lys, and His as one group (MKH), and Leu and Ile as another (IL), were studied [26]. Contrary to the single limiting AA theory, the results showed that, compared with the control group (saline), MP yield in groups of MKH, IL, and MKH+IL were improved by 84 g/d, 64 g/d, and 145 g/d, respectively [26]. The EAA requirements and limiting AA theory are still out for debate, and much more research is needed at the cellular and whole-animal levels [27].

## 3. Free and Peptide-Bound AAs Supply

### 3.1. EAAs Promote MP Synthesis

MP synthesis is affected by AA supply and AA profile [28,29], as well as the supply and type of energy [30,31]. Among them, EAAs play important roles in promoting MP synthesis in dairy cows. Increasing EAA supply to the MG is the basis of most dietary treatments to enhance MP content or MP yield. EAAs can increase MP synthesis through cell proliferation (cell viability and cell cycle progression) and activation of the mammalian target of rapamycin (mTOR) pathway in bovine mammary epithelial cells (BMECs) [32,33]. Some studies on the effects of EAAs on dairy cow MP synthesis are listed in Table 1.

### 3.2. Met

Met is the first limiting AA for ruminants [17]. Increasing the Met supply by feeding diets supplemented with RPM can increase the milk yield and MP yield [37,38,42,50]. The resistance to rumen degradation of many Met analogs have been studied. The D,L-2-hydroxy-4-(methylthio)-butanoic acid (HMB) is one Met analog which has been widely studiedis. The addition of HMB to the diet increased the dairy cows’ milk and MP yield [20]. However, the results of St-Pierre and Sylvester (2005) show that HMB seemed unable to meet the the needs of Met in MP synthesis of lactating dairy cows [51]. Isopropyl ester of HMB (HMBi) has also been used for Met supply to cows, with an increase observed in the MP yield and content [51,52,53]. The effect of Met on the synthesis of MP is multifaceted. Some research showed that an enhanced supply of RPM (0.9 g/kg of dry matter intake) increased the MP percentage and milk yield in early lactation of dairy cows along with a series of physiological changes, including increased dry matter intake, activity of protein kinase B (AKT), and upregulated glucose and AA transporter mRNA abundance, and transcripts of tRNases [54]. Supplementation of RPM at 0.08% dry matter of diet increased yields of milk (44.2 vs. 40.4 kg/d) and MP content (3.32% vs. 3.14%), and reduced the ketosis and retained placenta incidence [34]. In a study by Liu et al. (2019), lactating goats intravenously infused with an AA mixture with graded Met removal (100, 60, 30, or 0% of that in casein) showed that the MP yield dropped to 82, 78, and 69% of the 100% group, and the mTOR signaling pathway and overall AA catabolism seemed to be reasons for the changes [55]. In vitro experiments also demonstrated that Met could increase casein synthesis in BMECs by increasing cell proliferation and activating the mTOR signaling pathway in addition to being a substrate for MP synthesis [32,33,40].

### 3.3. Lys

Lys is another limiting AA for dairy cows. Supplementation with RPL increased the concentration of milk true protein [42]. The improvement of Lys and Met nutrition of dairy cows can increase the feed intake and MP content and yield [56]. In early lactation, the addition of 45 g of AA containing 5.6 g of RPM and 16.6 g of RPL increased the MP content and yield by 1 g/kg of milk and 37 g/d, respectively [57]. Supplementation of RPL prototype in dairy cows fed an RPL deficient diet also increased milk yields and trended to increase MP percent [58]. In a study by Lobos et al. (2021), supplemention with RPL (20 g absorbable Lys/d) to a corn protein-based diet increased the milk yield (1.1 kg/d) and milk true protein (50 g/d) of dairy cows [44]. In our previous study, the effects of Lys on MP synthesis and the mechanism of Lys uptake and catabolism in BMECs were investigated [59]. The results showed that compared with the 0, 0.5, and 2.0 mmol/L Lys groups, the cell viability and protein synthesis of the 1.0 mmol/L Lys group significantly increased by 17–47% and 7–23%, respectively, while the protein degradation decreased by 4–64%. Lys metabolism with [^U−14^C] L-Lys showed that the proportion of Lys used in protein synthesis, oxidation to carbon dioxide, synthesis of aspartate and His was 90%, 4%, 3% and 3%, respectively [59]. Similarly, a study by Morris and Kononoff (2020) found that the addition of RPL (24 g/d of digestible Lys) to a diet containing 5% hydrolyzed feather meal increased dairy cow protein synthesis and decreased protein degradation by increasing the N balance (25 to 16 g/d) and decreasing 3-methylhistidine (3.19 to 3.40 µM) [21]. Furthermore, Lys can also promote protein accretion by enhancing uptake by increasing sodium- and chloride-dependent neutral and basic AA transporter B^0,+^ (ATB^0,+^) expression and activating the mTOR and Janus kinase 2-signal transducer and activator of transcription 5 (JAK2-STAT5) signaling pathways by increasing the activity of mTOR (22%) and STAT5 (21%) [59].

### 3.4. BCAAs

Branched chain AAs (BCAAs), namely, Leu, Ile, and Val, play very important physiological and metabolic roles. In addition to being simple nutrition, BCAAs also can promote insulin release, enhance protein synthesis, and regulate the mTOR signaling pathway [60]. BCAAs are also important for MP synthesis in dairy cows. In addition to being the substrates for MP synthesis, BCAAs can also synthesize non EAAs required for MPs synthesis [61,62]. Recent studies have determined that, in addition to Met and Lys, BCAAs are potentially limiting for MP synthesis [23,45]. A deficiency in BCAAs may reduce casein gene transcription and inhibit the MP synthesis [43,63,64]. Furthermore, supplementation with Val promoted MP synthesis by upregulating AA transporters, changing EAA metabolism, and activating the mTOR signaling pathway [23]. The results of Huang et al. (2021) suggest that Ile and Leu can spare Lys and Met for milk protein synthesis [16].

### 3.5. AA Balance

It has been reported that the milk yield and true protein yield linearly increased with increasing dietary CP [65]. However, N efficiency can be improved by reducing dietary CP [65,66]. The quality of protein sources in the diet plays a key role in the growth, production, and reproduction of animals. Dietary AA imbalances can decrease synthesis of MP and reduce N metabolism efficiency. Some studies showed that the milk yield was inhibited when dairy cows were fed diets lacking limiting AAs, such as His, Met, and Lys [67,68,69]. Dietary crude protein (CP) with imbalanced AA cannot meet the needs of high-producing dairy cows. The addition of rumen-protected EAAs may balance dietary AAs and thereby improve lactation performance and N efficiency [27,70]. Supplementation with Met and Lys at the optimal ratio can reduce dietary CP amount, and improve the AA uptake of MG and the milk yield [20,56,71]. These responses are usually explained according to the theory of limiting AAs [72]. Balancing the EAA profile can increase the MP yield and metabolizable protein efficiency by increasing MG EAA uptake and decreasing AA catabolism [49,73]. The results of in vitro experiments have contributed to the same conclusion. MP gene expression in cultured BMECs or bovine mammary epithelial cell lines (MAC-Ts) was increased by an optimal concentration of individual EAAs and decreased by an excess or deficiency of any EAAs [39,74,75]. The optimal AA ratio promoted the MP synthesis by increasing AA transport into MECs, enhancing the regulation of insulin and the activity of the mTOR signaling pathway [64,76]. Thus, an appropriate ratio of EAAs can improve the AA balance and increase the synthesis of MP, leading to enhanced N utilization efficiency and dairy cow performance.

### 3.6. PBAAs Are Involved in MP Synthesis

FAAs are the main substrates for MP synthesis in the MG of lactating dairy cows. However, some free EAAs absorbed by the MG cannot meet the needs of MP synthesis [77,78]. These AAs that do not meet the requirements for MP synthesis appear to come from PBAA [79]. Backwell et al. (1996) analyzed AAs in the arterial plasma of lactating goats and found that 10–30% of the total AAs were in the form of PBAAs [78]. It has been reported that more than 25% of MP synthesis substrates come from rumen bypass peptides [80].

Results of Tagari et al. (2004) showed that several PBAAs were detected in the blood of lactating dairy cows, and a considerable number of the PBAA fraction was taken up by the MG [81]. The results from in vivo experiments with lactating dairy sheep and goats indicate that many EAAs are taken up by the MG from the circulation as PBAAs and utilized for protein synthesis [82,83]. Some studies showed that when dipeptides containing Met were used as a supplement of Met, casein synthesis was promoted in both cultured BMECs and MG explants [84,85,86]. Similarly, oligopeptides composed of Phe and Thr also increased *α_s1_-casein* gene mRNA abundance in BMECs compared with free Phe and Thr [87,88]. The study of Tagari et al. (2008) displayed that the peptide-bound EAAs in MG uptake accounts for 3.7% to 4.8% of the EAAs [79].

## 4. Mechanism of FAA and PBAA Promotion of MP Synthesis

MP synthesis uses FAAs and PBAAs taken up by the MG from blood flow as substrates. Furthermore, AAs (FAAs and PBAAs) promote MP synthesis by serving as signaling molecules.

### 4.1. Mechanism of the Promotion of MP Synthesis by FAA

#### 4.1.1. AA Transport Systems

The uptake of AA by the MG is conducted by different transporters located on the basolateral side of the plasma membrane in the MECs. The affinity of a transporter to an AA, as well as the number of AA transporters located in the plasma membrane, can affect the mammary net uptake of AAs. Multiple transporters for FAA have already been identified in mammary tissue [89,90]. These AA transporters play very important roles in MG AA uptake and MP synthesis [91]. Detailed information on AA transporters has been reviewed recently by Kandasamy et al. (2018) [92]. The transport system and substrates of AA transporters identified in bovine MG are shown in Table 2.

The AA transport systems in the MG can be regulated by many factors, such as hormones, physiological conditions, and substrates [88,99,100]. Activity and mRNA expression of sodium-coupled neutral AA transporter 2 (*SNAT2*) in rat mammary tissue increased at Days 12–16 of lactation, coinciding with the peak of milk production, and the addition of AAs could increase the *SNAT2* mRNA abundance in cultured lactating rat MG explants with lactogenic hormones present in the medium [100]. In addition, the mRNA abundance and protein of L-type AA transporter 1 (LAT1) is significantly higher in lactating bovine MG with high MP content (>3%) than in MG with low MP content (<3%); furthermore, the mTOR pathway may be the control point of LAT1 expression regulation [101]. It has also been reported that Met supplementation can enhance the transcription of neutral AA transporter (*SLC38A2*), high-affinity cationic transporter (*SLC7A1*), and *α_s1_-casein* [54]. The results of several studies have also shown that PBAA or Lys could increase *ATB^0,+^* mRNA abundance in both cultured BMECs and bovine mammary tissue explants [59,85,88].

#### 4.1.2. Role of AAs in Regulation of MP Synthesis

**Figure 1:**
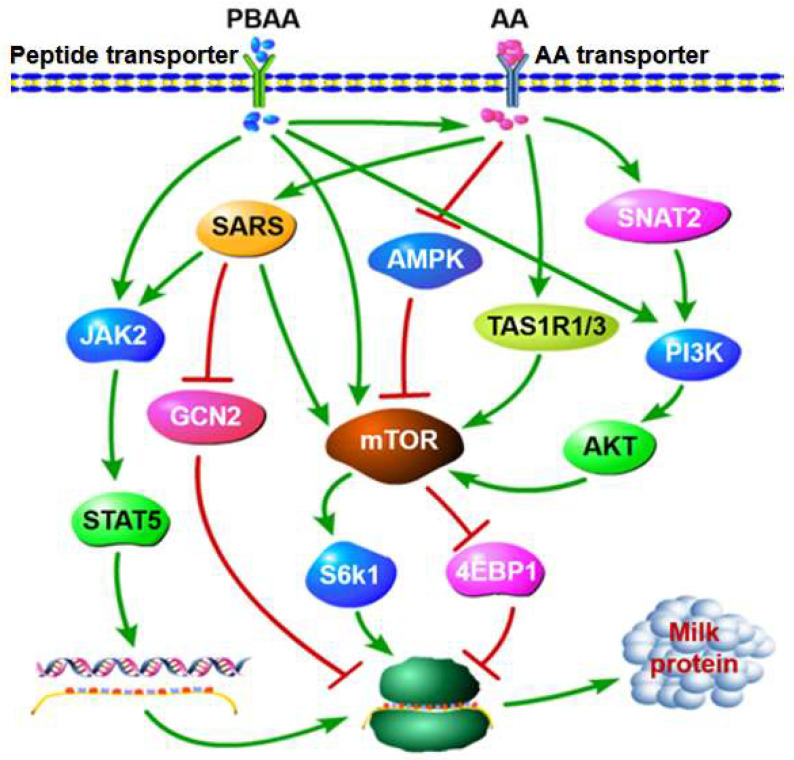
Signaling pathways of regulation of milk protein synthesis by free amino acids (FAAs) and peptide-bound amino acids (PBAA) in mammary epithelial cells (MECs).

The underlying molecular mechanism for controlling the MP yield of dairy cows is not completely clear. The role of AAs as signaling molecules, in addition to being substrates, in the regulation of protein synthesis is increasingly being recognized [102,103,104]. Direct signal transduction from AAs to the transcriptional and translational domains is involved in the synthesis of MP in BMECs [104,105]. Dietary AAs can regulate the DNA transcription and mRNA translation by activating the JAK2-STAT5, general control nonderepressible 2 kinase (GCN2), and mTOR signal transduction pathways (Figure 1) [101,106,107]. Supplementation with EAAs can increase MP synthesis by enhancing the mTOR and JAK2-STAT5 pathways, but inhibiting the GCN2 pathway in MAC-T cells and BMECs and the seryl-tRNA synthetase (SARS) mediates the positive regulation of MP synthesis by EAAs [33,103,108]. Edick et al. (2021) found that deprivation of Arg, Leu, and Lys in the culture medium activated the GCN2 pathway, which inhibited the translation of mRNA and reduced the synthesis of protein in BMECs [107]. Met has been reported to increase the *β-casein* mRNA abundance by activating the mTOR signaling pathway in the BMECs, and the AA taste 1 receptor member 1/3 (TAS1R1/TAS1R3) plays an important role in this process [40]. In addition, Met was reported to positively regulate MP and cell proliferation via the SNAT2-phosphatidylinositol-3-kinase (PI3K) pathway in the BMECs [32]. Other studies demonstrated that, compared with BMECs without Lys, 1.0 mmol/L Lys promoted BMEC MP synthesis by increasing the mRNA abundance and phosphorylated STAT5 and mTOR signaling proteins [59]. Furthermore, an increase in Val from 142 μg/mL to 156 μg/mL could also enhance MP synthesis of MAC-T by increasing the phosphorylation status of AKT, ribosomal protein S6 kinase beta-1 (S6k1), and mTORC1 [23]. In contrast, AA deficiency can reduce BMEC casein transcription and translation by inhibiting the JAK2/STAT5 and adenosine 5′-monophosphate-activated protein kinase (AMPK)/mTOR pathways, respectively [104]. Deficiency of BCAAs may decrease casein gene mRNA abundance and reduce MP production by upregulating eukaryotic initiation Factor 2B epsilon (eIF2Bε) and eukaryotic translation initiation Factor 2α (eIF2α) mediated by inactivation of mTOR [43,63,64]. However, abomasal infusion with EAAs for 5 days increased MP synthesis but did not affect the cell proliferation, protein gene mRNA abundance, ribosome biogenesis, or mTOR pathway activity in the MG of dairy cows [109]. The results of some studies suggest that the regulation of the unfolded protein response components that control endoplasmic reticulum biogenesis may contribute to long-term nutritional regulation of MP synthesis [109,110]. Incorporation of these concepts into MP response models will help to improve milk and MP yield predictions, increase postabsorptive N efficiency, and reduce N excretion by dairy cows.

Free AAs are taken up by MECs via AA transporters. PBAAs are transported into MECs by peptide transporters and hydrolysed to free AAs after entering the cells. In the cells, these AAs up-regulate the mammalian target of rapamycin (mTOR) signaling pathway by activation of seryl-tRNA synthetase (SARS), AA taste 1 receptor member 1/3 (TAS1R1/TAS1R3), phosphatidylinositol-3-kinase (PI3K-AKT) pathways and inhibition of adenosine 5′-monophosphate-activated protein kinase (AMPK). In addition, AA can activate Janus kinase 2-signal transducer and activator of transcription 5 (JAK2-STAT5) and inhibit general control nonderepressible 2 kinase (GCN2) pathway by activation of SARS; → activation;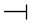 inhibition.

### 4.2. Mechanism of PBAAs Promoting MP Synthesis

#### 4.2.1. PBAA Transport Systems

PBAA may be taken up by MG in intact form or hydrolyzed to the corresponding FAA before absorption. Some studies have shown that PBAA is first hydrolyzed to FAA by small peptide hydrolase on the basement membrane of BMECs and then transported into MECs by the corresponding AA transporters [111,112]. Other studies found that small peptides can be absorbed in intact form [85,86]. The main small peptide transporters in epithelial cells are the peptide transporters 1 (PepT1) and 2 (PepT2). PepT1 plays an important role in the absorption of small peptides in the small intestine [113], and no expression of *PepT1* mRNA is observed in the MG of dairy cows [114]. The high-affinity, low-capacity transporter PepT2 is mainly expressed in the kidney tubules [115]. By using reverse transcriptase polymerase chain reaction (RT-PCR) and immunocytochemistry, *PepT2* mRNA and protein were detected in BMECs [87]. The identification of peptide transporters in the MG may therefore provide new insights into protein metabolism and synthesis by the gland. The expression of PepT2 in bovine MG has been reported to be upregulated by lactogenic hormones (i.e., prolactin, hydrocortisone, and insulin) and substrates (Met-Met, Phe-Phe, Phe-Thr, and Thr-Phe-Phe) [85,86,87,88]. The inhibition of PepT2 by diethylpyrocarbonate and PepT2 siRNA significantly reduced Met-Met uptake and decreased the Met-Met-stimulated synthesis increase in α_s1_-casein and β-casein in dairy cow mammary explants and BMECs [85,86]. Met-Met uptake in BMECs can also be inhibited by Met-Lys, Met-Leu, glycine (Gly)-Met, Lys-Lys, and Gly-Leu, indicating that these peptides are also substrates for peptide transporters in BMECs [86]. Furthermore, the PI3K-AKT pathway is involved in the regulation of β-alanyl-L-lysyl-Nε-7-amino-4-methyl-coumarin-3-acetic acid (β-Ala-Lys-AMCA, a model peptide) uptake in BMECs [116]. In summary, strong evidence has indicated that PepT2 plays a vital role in the transport of intact small peptides and MP synthesis. However, the transport kinetics of PepT2 for individual peptides remain unclear and need further study.

#### 4.2.2. Role of PBAAs in MP Synthesis

After uptake by MECs, PBAAs can promote MP synthesis and secretion, and this stimulation may be mediated by enhancing intracellular substrate availability, cell proliferation, and signaling pathways in BMECs [85,86,88].

First, PBAA can be used as a nutritional substrate for MP synthesis. The most likely mechanism is that PBAA transported by PepT2 is used for the synthesis of MP after intracellular hydrolysis to FAAs within MECs. This conclusion was confirmed by the research of Yang et al. (2015) [85], who found that replacement of 15% Met with Met-Met significantly increased *α_s1_-casein* mRNA abundance and protein expression in mammary explants, and inhibition of small peptide hydrolases (aminopeptidase N) by bestatin decreased the Met-Met-induced increase in α_s1_-casein synthesis.

Second, the uptake of PBAAs in intact form could reduce the competition of FAA uptake by AA transporters and thus promote MP synthesis. Several studies have shown that PBAA could enhance MP synthesis compared with an equivalent number of FAAs [85,86]. Evidence supports that the underlying mechanism may be that PBAAs could promote AA transporter expression and total uptake of some FAAs by reducing the competition for transporters during AA absorption. Zhou et al. (2015) showed that Phe-Phe could increase MP synthesis by promoting cationic AA transporter (*SLC6A14*) gene expression and the total uptake of some AAs (Lys, Leu, Ile, Phe, Val) [88]. Yang et al. (2015) also confirmed that Met-Met promoted MP synthesis by increasing the uptake of Met, Lys, His, Val, Leu, and Phe, and the mRNA abundance of neutral and basic AA transporters [85].

Third, PBAAs can also serve as signaling molecules in MP synthesis regulation (Figure 1). The promotion of casein synthesis by Met-Met may be mediated by the JAK2-STAT5 and mTOR pathways [85,86]. In the study of Wang et al. (2018), Met-Met promoted the activity of STAT5, JAK2, mTOR, 4EBP1, and S6k1 and thus increased cell proliferation, cell viability, and β-casein synthesis in BMECs [85]. In addition, the Met-Met-stimulated increase in cell viability and MP synthesis in BMECs was decreased by inhibiting phosphorylation of JAK2 and mTOR signaling pathways [86]. Furthermore, Chen et al. (2020) compared the effects of supplemental Met or Met-Met during pregnancy on Met-deficient mouse mammogenesis and lactogenesis, and found that, compared with Met, Met-Met promoted mammogenesis (42%) and lactogenesis (84%) more effectively by activating the PI3K-AKT signaling [117].

## 5. Conclusions and Perspectives

In summary, the synthesis of MP in dairy cow MG is a complex metabolic process. FAAs and PBAAs are very important substrates and signaling molecules for promoting MP synthesis. Increasing and balancing the AA (FAAs and PBAAs) supply to the MG is the basis of most dietary treatments to enhance MP content or yield. The uptake of sufficient quantities of well-balanced AAs is critical to improve MP yield in dairy cows. However, many areas remain to be studied, such as the determination of other AAs limiting MP synthesis, the regulation of individual AA or PBAA on MP synthesis, and the utilization mechanism of PBAAs in the MG.

## Figures and Tables

**Table 1:** Some recent studies on the effects of EAAs on MP synthesis in dairy cows.

EAA	Effects on MP Synthesis	Reference
Met	Increases in milk yield and MP yield	[24,34,35,36,37]
	Increases milk yield	[38]
	Increases in β-casein synthesis	[39,40]
	No effect on milk yield and MP	[41,42]
Lys	Increases in MP concentration	[22,35,43]
	Promotes β-casein synthesis	[39]
	Increases milk yield and milk true protein	[44]
Val	Increases in milk yield and MP synthesis	[23,43,45,46]
	Increase in casein mRNA abundance	[40]
	No effect on milk yield and MP	[24,41,42]
	Decrease in MP yield	[14]
Leu	Ppositively associated with milk yield and MP yield	[24,43]
	Promotes β-casein synthesis	[39]
	Decrease in MP yield	[14]
Ile	Increase in MP synthesis	[43,45]
	Decrease in MP yield	[14]
His	Increases in MP concentration and yield.	[24,35,47,48]
	Promotes β-casein synthesis	[39]
	Increases milk yield and tendes to increase MP yield	[21]
	Increases milk and MP yield	[49]
Phe	Positively associated with milk yield	[22]
Thr	Positively associated with milk yield	[24]
Arg	Positively associated with milk yield	[22]
	No effects on MP synthesis	[45]
Trp	Positively associated with milk yield	[24]

**Table 2:** Amino acid transporters identified in bovine mammary tissue.

Gene	Protein	Associated Transport System	Substrates	Reference
*SLC1A1*	EAAC1, EAAT3	X^−^ _AG_	Glutamate, aspartate	[93]
*SLC1A2*	GLT-1, EAAT2	X^−^ _AG_	Glutamate, aspartate	[93]
*SLC1A3*	GLAST, EAAT1	X^−^ _AG_	Glutamate, aspartate	[93]
*SLC1A4*	ASCT1, SATT	ASC	Alanine, serine, cysteine, and threonine	[88,93]
*SLC1A5*	ASCT2, AAAT	ASC	Neutral amino acid	[93]
*SLC3A2*	4F2hc	Heavy chain	Systems L, y^+^L, x_c_^−^ and asc with light subunits SLC7A5-8 and SLC7A10-11	[94,95]
*SLC6A1*	GAT1	GABA	Gamma-aminobutyric acid	[93]
*SLC6A6*	TauT	System β	Beta-alanine	[93]
*SLC6A9*	GlyT1	System Gly	Glycine	[96]
*SLC6A14*	ATB^0,+^	B^0,+^	Cationic amino acid	[88]
*SLC7A1*	CAT-1	y^+^	Cationic amino acid	[95,97]
*SLC7A2*	CAT-2	y^+^	Cationic amino acid	[88,95]
*SLC7A3*	CAT-3	y^+^	Cationic amino acid	[93,95]
*SLC7A5*	LAT1	L	Large neutral amino acid	[95,98]
*SLC7A7*	y^+^LAT1	y^+^L	Na+ indep.: cationic amino acids; Na+/large neutral amino acids	[93,95]
*SLC7A8*	LAT2	L	Neutral L-amino acids	[93,95]
*SLC16A10*	TAT1, MCT10	T	Aromatic amino acid	[88]
*SLC38A2*	SNAT2	A	Neutral amino acid	[93]
*SLC38A3*	SNAT3	N	Neutral amino acid	[93]

## Data Availability

Not applicable.
